# Self-Reported Physical Activity and Type 2 Diabetes in Adults Attending Primary Care: A Real-World Cross-Sectional Study of Cardiometabolic Risk

**DOI:** 10.3390/medicina62071367

**Published:** 2026-07-16

**Authors:** Peter Marián Kalanin, Ivan Uher

**Affiliations:** 1 Department of General Medicine, Faculty of Medicine, Pavol Jozef Šafárik University in Košice, 040 11 Kosice, Slovakia; peter.kalanin@upjs.sk; 2 Institute of Physical Education and Sport, Pavol Jozef Šafárik University in Košice, 040 11 Kosice, Slovakia; 3 MED-KAL s.r.o., 040 11 Kosice, Slovakia

**Keywords:** cardiometabolic risk, lifestyle behavior, logistic regression, observational study, physical activity, primary care, real-world data, risk stratification, type 2 diabetes mellitus

## Abstract

*Background and Objectives*: Physical inactivity is a major modifiable lifestyle factor associated with type 2 diabetes mellitus (T2DM). However, real-world primary care datasets often rely on pragmatic clinical estimates of physical activity (PA) and may lack HbA1c, fasting glucose, dietary data, diabetes duration, and detailed medication information. Therefore, PA–T2DM associations in routine care must be interpreted within the limitations of cross-sectional observational data. *Materials and Methods*: This cross-sectional observational study analyzed 863 adult patients from a real-world primary care cohort. Participants were categorized into low, moderate, and high PA groups according to self-reported habitual weekly activity levels estimated during routine physician interviews. PA categories were based on clinically meaningful weekly activity thresholds consistent with public health recommendations. The primary outcome was documented T2DM. Secondary variables included lipid profile, blood pressure, body mass index, waist circumference, smoking status, and statin therapy. Multivariable logistic regression models were used to assess associations between PA category and T2DM, with high PA as the reference group. *Results*: T2DM prevalence showed a graded inverse cross-sectional association across PA categories, being highest in the low PA group (29.7%), intermediate in the moderate PA group (16.7%), and lowest in the high PA group (8.9%) (*p* < 0.001). In the fully adjusted model, low PA was strongly associated with higher odds of T2DM compared with high PA (odds ratio [OR] 4.32, 95% confidence interval [CI] 2.61–7.15, *p* < 0.001). Moderate PA was also associated with higher odds of T2DM compared with high PA (OR 2.07, 95% CI 1.23–3.48, *p* = 0.006). LDL-C differed significantly across PA groups, with the lowest values observed in the high PA group, whereas most other cardiometabolic parameters were comparable. *Conclusions*: Lower self-reported PA was strongly associated with higher T2DM prevalence after multivariable adjustment in this real-world primary care cohort. These findings support routine PA assessment as part of lifestyle-based cardiometabolic risk stratification. Because dietary intake, HbA1c, diabetes duration, glucose-lowering medication details, and regulatory biomarkers were not available, causal and mechanistic conclusions cannot be drawn. Future studies should integrate PA, nutritional assessment, glycemic markers, medication data, and physiological regulatory measures to better characterize lifestyle-related diabetes risk.

## 1. Introduction

Type 2 diabetes mellitus (T2DM) is a major global health challenge and a central component of the cardiometabolic disease spectrum. The global burden of T2DM continues to increase, driven by population aging, sedentary lifestyles, excess adiposity, unhealthy dietary patterns, and broader lifestyle transitions [1,2,3]. Beyond hyperglycemia alone, T2DM is increasingly recognized as a systemic disorder embedded within a wider cardiometabolic phenotype characterized by insulin resistance, dyslipidemia, hypertension, central obesity, chronic low-grade inflammation, and vascular dysfunction [4,5,6]. This perspective supports examining T2DM within interacting physiological, nutritional, and behavioral processes rather than as an isolated glycemic outcome.

Physical activity (PA), together with diet, body-weight regulation, and pharmacological management, is a central component of T2DM prevention and cardiometabolic care. Dietary quality, energy balance, and nutrient composition are closely related to insulin sensitivity, glycemic control, lipid metabolism, and long-term diabetes risk [7,8,9]. PA is clinically relevant because it is associated with skeletal-muscle glucose uptake, GLUT4-mediated glucose transport, insulin sensitivity, body composition, lipid metabolism, blood pressure, inflammatory regulation, and functional health [10,11,12,13]. In primary care, even a simple PA assessment may therefore provide useful information for identifying patients with elevated cardiometabolic risk.

Large epidemiological studies and lifestyle intervention trials consistently demonstrate an inverse association between PA and T2DM risk, including evidence for dose–response relationships and meaningful reductions in diabetes incidence among high-risk individuals [14,15,16,17,18]. Even moderate PA levels have been associated with clinically relevant metabolic benefits, while higher PA levels may be linked to additional risk reduction [19,20]. However, despite this strong evidence base, several gaps remain relevant to routine clinical practice. Much of the available literature derives from controlled trials or population-based cohorts that may not fully capture the heterogeneity of patients encountered in everyday primary care settings [21]. In addition, many studies focus primarily on glycemic endpoints while giving less attention to the broader cardiometabolic profile, including lipid concentrations, blood pressure, body mass index, waist circumference, smoking status, and medication use within the same analytical framework [22,23].

Primary care represents a frontline setting for diabetes prevention, early risk identification, and cardiometabolic management. Patients commonly present with overlapping behavioral, nutritional, metabolic, and clinical risk factors, and clinical decisions occur under real-world conditions rather than controlled research environments [24]. Routine primary care datasets may therefore provide useful practice-based evidence on how PA relates to T2DM status and associated cardiometabolic characteristics in everyday clinical populations. At the same time, such datasets often lack detailed nutritional information, HbA1c, fasting glucose values, diabetes duration, diabetes severity, detailed medication data, alcohol use, and nutritional counseling. These limitations are particularly relevant for clinical interpretation and should be explicitly considered when evaluating PA–T2DM associations within a broader lifestyle and cardiometabolic framework.

The relationship between PA and T2DM is also subject to possible reverse causality, particularly in cross-sectional primary care datasets. Although regular PA may contribute to improved insulin sensitivity, body-weight regulation, lipid metabolism, and vascular function, individuals with better baseline health, lower symptom burden, fewer diabetes-related complications, and greater physiological reserve may also be more capable of sustaining higher PA levels. Therefore, lower PA may function both as a lifestyle exposure and as a clinical marker of poorer underlying health status.

The present study aimed to evaluate the association between self-reported PA and documented T2DM in a real-world primary care cohort of 863 adult patients. Secondary objectives were to assess whether PA categories differed across key cardiometabolic variables, including lipid profile, blood pressure, body mass index, waist circumference, smoking status, and statin therapy. We hypothesized that lower PA would be associated with higher T2DM prevalence and a less favorable cardiometabolic profile.

This study extends conventional PA–diabetes epidemiology by examining self-reported PA in relation to documented T2DM prevalence and cardiometabolic characteristics in routine primary care data within a lifestyle and cardiometabolic risk framework. Because dietary intake, HbA1c, fasting glucose values, diabetes duration, glucose-lowering medication details, insulin sensitivity, inflammatory markers, autonomic balance, and recovery dynamics were not directly measured, nutritional, causal, and mechanistic interpretations should be considered exploratory and hypothesis-generating.

## 2. Materials and Methods

### 2.1. Study Design and Reporting

This cross-sectional observational study analyzed real-world primary care data to evaluate the association between self-reported PA and documented T2DM. The study was designed and reported in accordance with the STROBE (Strengthening the Reporting of Observational Studies in Epidemiology) statement. Study objectives, exposure definition, outcome specification, covariate selection, and statistical modeling strategy were defined prior to analysis. Because of the cross-sectional design, the study was not intended to determine causal effects of PA on T2DM risk.

### 2.2. Setting and Data Source

Data were retrospectively obtained from a routine primary care database reflecting standard clinical practice in a general medicine setting in Slovakia. The dataset included consecutive adult patients evaluated during routine outpatient visits. Demographic, anthropometric, clinical, lifestyle, and biochemical variables were extracted from medical records collected as part of standard care. Measurements were performed according to routine clinical protocols rather than research-specific procedures, thereby enhancing ecological validity and improving the applicability of findings to real-world primary care populations. Detailed dietary information, alcohol use, HbA1c, fasting glucose values, diabetes duration, diabetes severity, medication adherence, and detailed glucose-lowering medication data were not systematically available in the routine primary care database.

### 2.3. Study Population

The analytical sample comprised 863 adult patients aged 18 years or older. Although this analysis was derived from the same routine primary care database as previous related studies, the present diabetes-specific analytical cohort was defined according to available PA classification, documented T2DM status, and complete cardiometabolic and treatment-related variables required for the current regression models. Therefore, PA-group distributions may differ from previous analyses that used different primary outcomes, covariate requirements, or exclusion criteria. Eligible participants were required to have available PA classification, documented T2DM status, and complete core cardiometabolic variables, including lipid profile, blood pressure, and anthropometric measurements. Patients with missing exposure or outcome data, implausible clinical values identified during quality control procedures, or duplicate and internally inconsistent records were excluded. A complete-case analytical strategy was adopted to minimize uncertainty associated with imputation in multivariable models.

### 2.4. Exposure Definition: Physical Activity

PA was categorized into three clinically meaningful levels based on patient self-report during routine physician interviews. During clinical evaluation, physicians asked patients whether they performed regular habitual PA, how many days per week they were usually active, the approximate duration of each activity session, and the usual type and intensity of activity. Weekly PA volume was estimated by multiplying the reported frequency by the average duration of activity sessions and was expressed as minutes per week. Low PA represented activity levels below recommended thresholds, corresponding to less than 150 min of moderate-equivalent activity per week. Moderate PA reflected activity levels between 150 and 300 min per week, while high PA represented activity exceeding 300 min per week.

These categories were selected because they are consistent with commonly used public health thresholds and WHO recommendations that adults undertake at least 150–300 min of moderate-intensity aerobic PA per week, or an equivalent amount of vigorous-intensity activity. PA classification was derived from routine clinical assessment rather than standardized questionnaires, IPAQ or GPAQ, accelerometry, wearable devices, or formal MET-minute calculations. Therefore, PA categories should be interpreted as pragmatic real-world clinical estimates of habitual weekly activity rather than precise measures of energy expenditure. Information on PA intensity, type, frequency, sedentary behavior, occupational activity, and seasonal variation was not systematically available.

### 2.5. Outcome Definitions

The primary outcome was documented T2DM, defined as a physician-recorded diagnosis in the medical file rather than newly classified diabetes based on study-specific glycemic testing. HbA1c values, fasting glucose values, diabetes duration, and detailed glucose-lowering medication information were not systematically available and therefore could not be included in outcome classification or subgroup analyses. Secondary outcomes included cardiometabolic variables selected to characterize multidimensional metabolic risk. These comprised low-density lipoprotein cholesterol (LDL-C), high-density lipoprotein cholesterol (HDL-C), triglycerides (TG), systolic blood pressure (SBP), diastolic blood pressure (DBP), body mass index (BMI), and waist circumference.

### 2.6. Covariates and Confounding Control

Potential confounders were selected a priori based on established associations with both PA behavior and diabetes risk. Included covariates comprised age, sex, smoking status, statin therapy, body mass index, and systolic blood pressure. These variables were incorporated into adjusted regression models to reduce measured confounding and improve interpretability of the observed association between PA and T2DM. Additional clinically relevant covariates, including detailed dietary information, alcohol use, socioeconomic status, diabetes duration, HbA1c, fasting glucose values, medication adherence, comorbidities, and detailed glucose-lowering therapy, were not available and could not be included in the adjusted models.

### 2.7. Data Preprocessing and Quality Assurance

Data completeness and internal consistency were evaluated prior to analysis. Continuous variables were inspected for outliers and implausible distributions using range plausibility checks and graphical inspection. Clinically implausible values were excluded. Categorical variables were cross-validated for logical consistency, including agreement between diagnosis and available treatment-related variables. No imputation procedures were performed, and analyses were restricted to complete cases to preserve transparency and avoid model uncertainty introduced by missing data estimation.

### 2.8. Statistical Analysis

Descriptive statistics were used to characterize the study population. Continuous variables are presented as mean ± standard deviation (SD), while categorical variables are reported as counts and percentages. Differences across PA categories were assessed using one-way analysis of variance (ANOVA) for continuous variables and χ^2^ tests for categorical variables. Assumptions of normality and homogeneity of variance were evaluated prior to analysis. Where assumptions for parametric testing were not adequately met, non-parametric analyses, including the Kruskal–Wallis test for continuous variables, were considered as sensitivity checks. For T2DM prevalence across PA categories, 95% confidence intervals were calculated for proportions to improve the precision and interpretability of prevalence estimates. Graphical presentation was added using a bar chart for T2DM prevalence across PA groups and a forest plot for logistic-regression odds ratios.

Additional cardiometabolic risk indices were considered to provide a more integrated assessment of metabolic risk beyond isolated lipid and anthropometric parameters. The TG/HDL-C ratio was calculated descriptively as triglycerides divided by HDL-C, using group mean values expressed in mmol/L. Because this ratio was derived from aggregate group-level values rather than individual-level patient data, it was interpreted as an approximate descriptive index only. TyG-based indices, including TyG-WHtR, were not calculated because fasting glucose values were not systematically available in the routine primary care dataset. WHtR was not included unless valid height data were available.

### 2.9. Logistic Regression Modeling

Associations between self-reported PA and documented T2DM were examined using multivariable logistic regression. Three sequential models were constructed. Model 1 evaluated the crude association between self-reported PA and documented T2DM. Model 2 adjusted for age and sex. Model 3 included additional adjustment for body mass index, smoking status, statin therapy, and systolic blood pressure. High PA served as the reference category. Results are reported as odds ratios (ORs) with corresponding 95% confidence intervals (CIs) and two-sided *p*-values.

### 2.10. Model Diagnostics

Regression assumptions were evaluated before interpretation of model results. Multicollinearity was assessed using variance inflation factors (VIFs). Model fit was evaluated using appropriate logistic-regression diagnostics, including the Hosmer-Lemeshow goodness-of-fit test where applicable. Influential observations were examined through residual diagnostics. Stability of regression coefficients across nested models was assessed to evaluate the internal consistency of the findings.

### 2.11. Sensitivity Analyses

Sensitivity analyses were performed to assess the internal stability of the observed associations. These included comparison of crude and adjusted models, evaluation of attenuation of effect estimates following covariate adjustment, and examination of consistency in the direction and magnitude of associations across models. Given the cross-sectional design, sensitivity analyses focused on internal stability rather than causal inference.

### 2.12. Statistical Thresholds

Statistical significance was defined as *p* < 0.05, with exact *p*-values reported where appropriate. All statistical tests were two-sided.

### 2.13. Ethical Considerations

This study was conducted in accordance with the principles of the Declaration of Helsinki (2013 revision). The analysis was based on anonymized, routinely collected clinical data, and no identifiable personal information was used. According to national regulations, separate formal ethical approval was not required for retrospective analyses of anonymized data.

### 2.14. Data Availability

Due to the clinical and real-world nature of the dataset, individual-level data are not publicly available. Aggregated data may be provided upon reasonable request to the corresponding author.

## 3. Results

### 3.1. Study Population and Baseline Characteristics

The final study population comprised 863 adult patients, stratified by self-reported PA into low (*n* = 263), moderate (*n* = 341), and high (*n* = 259) PA groups.

Baseline characteristics are summarized in Table 1.

Age differed modestly across groups, with a slightly higher mean age observed in the moderate PA group compared with the low and high PA groups (*p* = 0.041). Sex distribution was comparable across PA categories (*p* = 0.911). Most cardiometabolic parameters, including BMI, waist circumference, systolic and diastolic blood pressure, HDL-C, and triglycerides, did not differ significantly between groups (all *p* > 0.05). In contrast, LDL-C showed a statistically significant difference, with lower mean values observed in the high PA group (*p* = 0.002).

T2DM prevalence differed markedly across PA categories, with the highest prevalence observed in the low PA group (29.7%), followed by the moderate PA group (16.7%), and the lowest prevalence in the high PA group (8.9%) (*p* < 0.001). Smoking status and statin therapy also differed significantly between groups (*p* = 0.015 and *p* = 0.038, respectively). Overall, baseline cardiometabolic characteristics were largely comparable across PA categories, while differences in T2DM prevalence and LDL-C suggested that self-reported PA was related to selected metabolic-risk indicators in this cohort.

### 3.2. T2DM Prevalence Across PA Levels

A graded inverse cross-sectional pattern in T2DM prevalence was observed across PA categories. T2DM was present in 78 of 263 participants in the low PA group (29.7%, 95% CI 24.5–35.4), 57 of 341 participants in the moderate PA group (16.7%, 95% CI 13.1–21.0), and 23 of 259 participants in the high PA group (8.9%, 95% CI 6.0–13.0). The between-group difference was statistically significant (*χ^2^ p* < 0.001).

**Figure 1:**
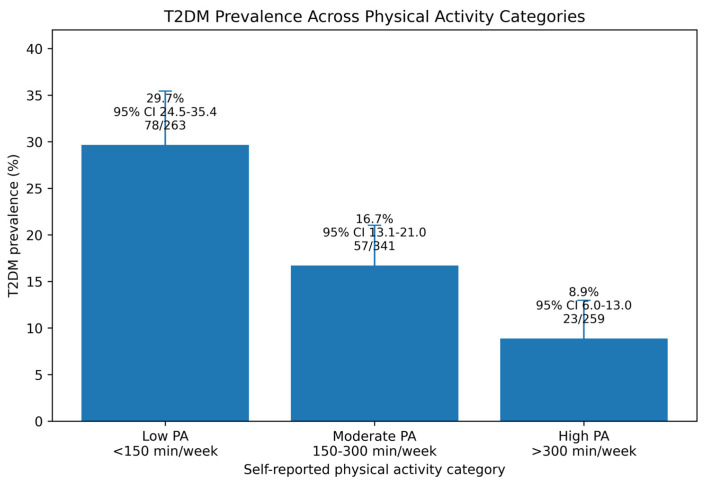
Prevalence of type 2 diabetes mellitus across self-reported physical activity categories. Error bars represent 95% confidence intervals for prevalence estimates. T2DM prevalence was highest in the low physical activity group, intermediate in the moderate physical activity group, and lowest in the high physical activity group, indicating a graded inverse cross-sectional association between physical activity level and documented type 2 diabetes mellitus. PA, physical activity; T2DM, type 2 diabetes mellitus; CI, confidence interval.

In absolute terms, T2DM prevalence was 20.8 percentage points higher in the low PA group compared with the high PA group, corresponding to an approximately 3.3-fold higher crude prevalence. As shown in Figure 1 and Table 2, T2DM prevalence progressively decreased with increasing levels of self-reported PA, consistent with a graded inverse cross-sectional association between PA and T2DM.

### 3.3. Cardiometabolic Profile According to PA

Consistent with the baseline analysis, most cardiometabolic parameters did not differ significantly across PA groups. BMI, waist circumference, systolic and diastolic blood pressure, HDL-C, and triglycerides remained comparable between categories.

LDL-C differed significantly across PA categories, with the lowest mean value observed in the high PA group. In contrast, HDL-C and triglycerides did not differ significantly across groups. This suggests that the lipid-related difference observed in this cohort was driven primarily by LDL-C rather than by a broader uniform difference across all lipid parameters. However, because statin therapy also differed across PA groups, LDL-C findings should be interpreted cautiously and may reflect a combination of PA-related metabolic factors, treatment patterns, and residual confounding.

The lack of significant differences in most measured baseline variables suggests that the observed variation in T2DM prevalence was not fully explained by the measured traditional cardiometabolic characteristics.

### 3.4. Additional Cardiometabolic Risk Index

To provide a more integrated lipid-based estimate of cardiometabolic risk, the TG/HDL-C ratio was additionally considered. Based on the group mean values presented in Table 1, the approximate TG/HDL-C ratio was 1.34 in the low PA group, 1.36 in the moderate PA group, and 1.32 in the high PA group. Thus, unlike LDL-C, the TG/HDL-C ratio did not show a clear graded difference across self-reported PA categories.

Because this calculation was based on aggregate group mean values rather than individual-level patient ratios, it should be interpreted descriptively and not as a separate inferential statistical test. TyG-WHtR was not calculated because fasting glucose values were not systematically available in the dataset.

### 3.5. Logistic Regression Analysis

Multivariable logistic regression models were used to examine whether the association between self-reported PA and documented T2DM persisted after adjustment for potential confounding variables.

In the crude model, compared with the high PA group used as the reference category, moderate PA was associated with a 2.06-fold higher odds of T2DM (95% CI 1.23–3.44, *p* = 0.006), while low PA was associated with a 4.33-fold higher odds of T2DM (95% CI 2.62–7.16, *p* < 0.001).

After adjustment for age and sex, the association remained largely unchanged. Moderate PA was associated with an odds ratio (OR) of 2.06 (95% CI 1.23–3.45, *p* = 0.006), and low PA with an OR of 4.31 (95% CI 2.61–7.13, *p* < 0.001).

In the fully adjusted model, which included age, sex, BMI, smoking status, statin therapy, and systolic blood pressure, the associations remained statistically significant and largely unchanged in magnitude. Moderate PA was associated with a 2.07-fold higher odds of T2DM (95% CI 1.23–3.48, *p* = 0.006), whereas low PA was associated with a 4.32-fold higher odds of T2DM (95% CI 2.61–7.15, *p* < 0.001; Table 3).

**Figure 2:**
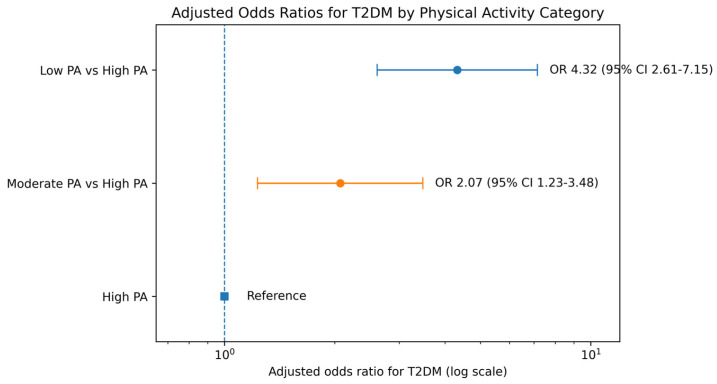
Forest plot showing adjusted odds ratios for documented type 2 diabetes mellitus according to self-reported PA category. High PA served as the reference group. The fully adjusted model included age, sex, BMI, smoking status, statin therapy, and systolic blood pressure. Compared with high PA, both moderate and low PA were associated with significantly higher odds of type 2 diabetes mellitus. OR, odds ratio; CI, confidence interval; PA, physical activity; T2DM, type 2 diabetes mellitus; BMI, body mass index.

These findings indicate that the relationship between lower self-reported PA and higher odds of T2DM persisted after adjustment for demographic and cardiometabolic variables. Minimal attenuation of effect estimates across models suggests that the observed association was stable after sequential adjustment. The fully adjusted odds ratios are graphically presented in Figure 2, while the full sequence of crude, age-and sex-adjusted, and fully adjusted models is reported in Table 3.

### 3.6. Summary of Results

Overall, T2DM prevalence increased progressively from high to low levels of self-reported PA. Participants in the low PA group had approximately fourfold higher odds of T2DM compared with the high PA group, and this association remained stable after multivariable adjustment. Most measured baseline cardiometabolic characteristics were comparable across PA groups, with LDL-C representing the only individual metabolic marker that differed significantly. The additional descriptive TG/HDL-C ratio derived from group mean values was similar across PA categories and did not show a clearer graded pattern than LDL-C. These findings support a graded inverse cross-sectional association between self-reported PA level and T2DM prevalence in this cohort, while suggesting that the measured lipid-derived indices only partly captured the observed PA–T2DM pattern.

## 4. Discussion

### 4.1. Principal Findings

In this real-world primary care cohort of 863 adults, lower levels of self-reported PA were strongly associated with a higher prevalence of T2DM. A graded inverse cross-sectional pattern was observed across PA categories, with T2DM prevalence increasing from 8.9% in the high PA group to 29.7% in the low PA group. Logistic regression analysis showed that participants with low PA had more than fourfold higher odds of T2DM compared with highly active individuals, and these associations remained stable after adjustment for demographic and cardiometabolic covariates.

An important finding is that most measured cardiometabolic parameters, including BMI, waist circumference, systolic and diastolic blood pressure, HDL-C, and triglycerides, did not differ significantly across PA groups. This indicates that the strong PA–T2DM association was not simply mirrored by broad differences in standard anthropometric, hemodynamic, or lipid markers. One interpretation is that self-reported PA may capture clinically relevant information about functional capacity, lifestyle pattern, health status, or metabolic vulnerability that is not fully represented by routine clinical markers. Another possibility is that unmeasured variables, including glycemic control, diabetes duration, medication patterns, diet, socioeconomic factors, and comorbidities, contributed to the observed association.

### 4.2. Comparison with Existing Evidence

The present findings are consistent with previous epidemiological studies reporting an inverse relationship between PA and T2DM risk. Large population-based cohorts have shown that insufficient PA is associated with impaired glucose regulation, increased insulin resistance, and elevated diabetes incidence. Lifestyle intervention trials have further demonstrated that increased PA can substantially reduce diabetes risk, particularly when combined with dietary modification and weight control. These observations align with landmark diabetes-prevention trials reported by Knowler et al. and Tuomilehto et al. [14,15].

Recent population-based and cohort evidence is consistent with the relationship between higher PA and lower T2DM risk. Yang et al. reported that different levels of PA were associated with the risk of developing T2DM among adults with prediabetes [25]. Zhou et al. further showed that PA domains and inflammatory dietary patterns may be independently and jointly associated with T2DM risk [26]. In addition, recent evidence has highlighted that the health effects of PA may differ according to context, intensity, and type of activity, particularly in individuals with T2DM [27].

The magnitude of cross-sectional association observed in the present cohort was relatively strong compared with many population studies. This may reflect the real-world nature of the sample, which includes adults receiving routine primary care and likely presenting with multiple coexisting metabolic risk factors. Unlike highly selected trial populations, primary care cohorts may better capture the complexity of cardiometabolic disease in everyday clinical practice.

The present study contributes to the literature by examining PA within a broader lifestyle and cardiometabolic context rather than focusing solely on documented T2DM status. The integration of anthropometric, hemodynamic, lipid, smoking, and treatment-related variables provides a more comprehensive perspective on metabolic risk patterns observed in routine care.

### 4.3. Physiological Mechanisms

Several biologically plausible physiological pathways may help contextualize the observed association between lower PA and higher T2DM prevalence. PA has been linked to reduced skeletal-muscle glucose uptake, impaired mitochondrial efficiency, and decreased insulin sensitivity. Reduced energy expenditure may further promote visceral adiposity, systemic inflammation, and metabolic dysregulation. These pathways are supported by classical metabolic research linking exercise, skeletal-muscle glucose uptake, insulin action, and inflammatory regulation [10,11,12,13].

PA may also influence glucose homeostasis through improved endothelial function, enhanced skeletal-muscle oxidative capacity, favorable lipid metabolism, and reduced inflammatory signaling. Recent systematic reviews and consensus statements further support the role of PA in improving glycemic control, metabolic regulation, and diabetes-related outcomes in adults with T2DM [28,29,30,31].

The lower LDL-C observed in the high PA group may have several possible explanations. Regular PA may influence lipid metabolism through improved skeletal-muscle oxidative capacity, enhanced lipid utilization, reduced inflammation, improved insulin sensitivity, and favorable effects on hepatic lipid handling. These pathways may operate partly independently of BMI, which did not differ across PA groups in the present cohort. However, the LDL-C finding should not be interpreted as a direct causal effect of PA. Statin therapy differed between PA groups, and detailed information on statin dose, adherence, treatment duration, diet, alcohol use, and diabetes medication was not available. Therefore, the observed LDL-C difference may reflect both biological and treatment-related factors.

The persistence of the association after adjustment for BMI, smoking status, blood pressure, and statin therapy suggests that self-reported PA may capture lifestyle-related metabolic risk information not fully represented by conventional cardiometabolic variables. However, the present study did not directly assess insulin sensitivity, inflammatory markers, autonomic regulation, mitochondrial function, HbA1c, fasting glucose dynamics, or diabetes duration. Therefore, no mechanistic conclusions can be drawn from the current data.

### 4.4. Clinical Lifestyle and Cardiometabolic Context

Nutrition and PA represent complementary components of lifestyle-based T2DM prevention and cardiometabolic risk reduction. Dietary quality, total energy intake, body-weight regulation, and macronutrient composition may interact with PA in shaping insulin sensitivity, lipid metabolism, adiposity, and long-term metabolic risk [7,8,9]. Therefore, PA should not be interpreted as an isolated lifestyle factor, but as one component of a broader lifestyle and cardiometabolic framework.

In the present real-world primary care dataset, detailed dietary information was not systematically available. This limitation is important for clinical interpretation, because diet quality, caloric intake, macronutrient composition, alcohol use, and nutritional counseling may influence both PA behavior and T2DM status. Future studies should integrate self-reported PA assessment with dietary intake, body composition, glycemic markers, medication data, and physiological biomarkers to provide a more complete lifestyle-based model of diabetes risk.

### 4.5. Life-Course and Developmental Context of Metabolic Risk

Although the present study focused on adult self-reported PA and documented T2DM status in routine primary care, metabolic risk should also be interpreted within a broader life-course framework. T2DM susceptibility may reflect not only adult lifestyle behaviors, adiposity, and cardiometabolic risk factors, but also earlier developmental influences that shape metabolic regulation across the lifespan. Intrauterine exposures, including gestational diabetes mellitus, maternal hyperglycemia, and pregnancy-related therapeutic interventions, may influence fetal and neonatal metabolic, cardiovascular, and neurodevelopmental trajectories and may contribute to later vulnerability to insulin resistance and metabolic dysfunction [31].

Babović et al. reviewed the complex relationship between gestational diabetes mellitus, antenatal corticosteroid therapy, fetal physiology, and neonatal outcomes, emphasizing that maternal-fetal metabolic conditions may have clinically relevant short- and long-term implications [31]. From this perspective, the observed association between lower PA and higher T2DM prevalence in the present adult primary care cohort should not be interpreted as arising solely from current behavior. Rather, adult PA may interact with pre-existing biological susceptibility, accumulated metabolic burden, and life-course determinants of cardiometabolic health. However, because data on birth history, maternal diabetes, intrauterine exposures, prematurity, and antenatal corticosteroid exposure were not available in the present dataset, this interpretation remains contextual and hypothesis-generating.

### 4.6. Hypothesis-Generating Regulatory Perspective

Beyond conventional metabolic pathways, self-reported PA may also reflect broader behavioral and physiological capacity, including recovery, stress regulation, autonomic flexibility, and the ability to sustain lifestyle engagement. These regulatory mechanisms were not directly measured in the present study and should therefore be considered hypothesis-generating only. Future studies incorporating heart rate variability, stress biomarkers, recovery dynamics, and objective PA monitoring are needed to test whether regulatory efficiency contributes to the relationship between PA and cardiometabolic outcomes.

The additional consideration of the TG/HDL-C ratio provides a more integrated lipid-based perspective than isolated TG and HDL-C values alone. In the present cohort, however, the approximate TG/HDL-C ratio derived from group mean values was similar across PA categories and did not show a clearer graded pattern than LDL-C. This suggests that the strong inverse association between PA and T2DM prevalence was not fully explained by the available lipid-based cardiometabolic indicators. Rather, PA may reflect broader lifestyle, functional, metabolic, or health-status differences that were only partly captured by the measured clinical variables.

### 4.7. Integrated Cardiometabolic Perspective

A notable finding of this study is the clustering of T2DM prevalence with selected metabolic differences despite otherwise comparable baseline characteristics. This pattern is consistent with the concept of cardiometabolic disease as an integrated network rather than a collection of isolated risk factors.

Within this framework, self-reported PA may be associated with interacting pathways related to glucose metabolism, lipid regulation, vascular function, inflammation, and cardiometabolic risk. Recent evidence suggests that lower PA may be associated with a broader cardiometabolic phenotype characterized by impaired glucose regulation, increased cardiovascular risk, and metabolic dysfunction [32,33,34].

The present findings align with contemporary views of cardiometabolic health as a dynamic interaction between lifestyle behavior, physiology, and long-term metabolic adaptation. Rather than operating through a single pathway, PA may reflect, or be associated with, distributed processes across multiple biological systems. However, because the present study was cross-sectional, this interpretation remains observational.

### 4.8. Novelty and Contribution

The study contributes to the literature in several important ways. First, it provides real-world evidence from a primary care cohort, thereby enhancing clinical relevance and external validity. Second, it evaluates self-reported PA in relation to multiple cardiometabolic variables rather than focusing exclusively on T2DM status. Third, it shows a strong graded cross-sectional association between PA category and T2DM prevalence across progressively lower activity levels.

An additional contribution lies in the interpretation of PA as part of a broader lifestyle and cardiometabolic risk framework. The persistence of the observed association after multivariable adjustment suggests that self-reported PA may capture clinically relevant lifestyle-related risk information not fully represented by the measured standard clinical markers.

### 4.9. Clinical Implications

The findings support the potential value of routine assessment of PA in primary care settings. Even simple categorization of PA levels may provide clinically meaningful information for identifying individuals with elevated metabolic risk. Importantly, PA should not be interpreted only as a modifiable behavior or intervention target. In routine clinical practice, low PA may also function as a practical marker of reduced health status, lower physiological reserve, symptom burden, or impaired capacity for sustained lifestyle engagement. Thus, PA assessment may provide clinically relevant information not only about behavior itself, but also about the broader functional and cardiometabolic condition of the patient.

Given the strong cross-sectional association observed between low PA and T2DM prevalence, self-reported PA assessment may be useful as part of practical cardiometabolic risk stratification. Clinicians may benefit from integrating PA screening into preventive care strategies alongside traditional metabolic risk assessment. However, clinical recommendations should be interpreted within the broader context of nutrition, body-weight regulation, glycemic status, medication use, and other lifestyle-related risk factors.

### 4.10. Strengths and Limitations

This study has several strengths, including the use of a real-world primary care cohort, a relatively large sample size, and the inclusion of multiple cardiometabolic variables. The stability of results across progressively adjusted regression models supports the internal consistency of the observed associations.

Several limitations should also be acknowledged. The cross-sectional design precludes causal inference and limits interpretation regarding the directionality of the observed associations. Therefore, the present findings cannot determine whether low PA contributed to higher T2DM prevalence, whether poorer metabolic health limited the capacity for PA, or whether both pathways operated simultaneously. Accordingly, self-reported PA in this study should be interpreted both as a lifestyle exposure and as a potential marker of underlying health status.

The study was conducted in a Slovak primary care cohort, and the findings may not be directly generalizable to other countries, healthcare systems, ethnic groups, socioeconomic settings, or populations with different PA patterns, dietary habits, diabetes management practices, and cardiometabolic risk profiles. External validation in broader European and international primary care cohorts is warranted.

The dataset also did not include life-course or developmental variables, such as birth weight, prematurity, maternal gestational diabetes, intrauterine exposure to hyperglycemia, or antenatal corticosteroid therapy. Therefore, the study could not evaluate whether early-life metabolic programming contributed to adult T2DM susceptibility or modified the association between PA and T2DM prevalence.

Dietary intake, diet quality, caloric intake, macronutrient composition, alcohol use, and nutritional counseling were not systematically available and therefore could not be included in the analysis. This limitation is particularly relevant for a clinical interpretation of lifestyle-related risk and limits conclusions regarding diet–PA interactions within the broader lifestyle framework.

PA classification was based on routine clinical self-report rather than a validated PA questionnaire, accelerometry, or wearable-device data. Information on PA intensity, type, frequency, sedentary behavior, occupational activity, and seasonal variation was also unavailable.

The absence of HbA1c, fasting glucose values, diabetes duration, diabetes severity, detailed glucose-lowering medication data, dietary intake, alcohol use, and socioeconomic information represents a major limitation. These variables may influence both PA behavior and T2DM status and could not be included in the adjusted models. Therefore, residual confounding and reverse causality cannot be excluded.

Additional integrated cardiometabolic indices were limited by the variables available in the routine primary care dataset. The TG/HDL-C ratio could be considered only descriptively from aggregate group mean values, and individual-level TG/HDL-C ratios were not analyzed unless individual patient lipid data were available. TyG-based indices, including TyG-WHtR, could not be calculated because fasting glucose values were not systematically available. WHtR was also not included unless valid height data were available.

Residual confounding cannot be excluded. Unmeasured factors such as dietary behavior, socioeconomic status, alcohol use, sleep quality, medication adherence, comorbidities, and psychological stress may have influenced the observed associations.

Regulatory mechanisms remain hypothesis-generating because physiological markers such as heart rate variability, recovery dynamics, autonomic regulation, interoceptive measures, and stress biomarkers were not measured.

### 4.11. Future Directions

Future research should incorporate longitudinal study designs to determine temporal relationships between PA and T2DM risk. Objective PA monitoring, combined with dietary assessment, glycemic biomarkers, medication data, body-composition measures, and physiological indicators, may provide deeper insight into the pathways linking lifestyle behavior with metabolic outcomes.

Further longitudinal investigations should also consider life-course variables, including maternal metabolic status, gestational diabetes, prematurity, birth weight, and relevant antenatal exposures, to clarify how early developmental factors interact with adult PA, adiposity, and cardiometabolic risk in shaping T2DM susceptibility.

Future prospective longitudinal studies should include objective PA assessment, validated PA questionnaires, HbA1c, fasting glucose, diabetes duration, medication data, dietary assessment, socioeconomic variables, and physiological biomarkers. Such studies are needed to determine whether PA independently predicts incident T2DM or whether self-reported PA primarily reflects broader baseline health status, metabolic reserve, and functional capacity.

Longitudinal research should further consider whether autonomic flexibility, stress-related physiology, recovery dynamics, or interoceptive processes help explain the relationship between PA engagement and metabolic outcomes. Such studies could clarify whether regulatory efficiency represents a measurable pathway linking lifestyle behavior, metabolic regulation, and diabetes risk.

## 5. Conclusions

In this real-world primary care cohort, lower levels of self-reported PA were strongly associated with a higher prevalence of T2DM. A graded inverse cross-sectional association was observed across PA categories, and the association remained stable after adjustment for demographic and cardiometabolic variables.

The observed PA–T2DM association was not fully explained by the measured clinical variables included in the present analysis. However, because of the cross-sectional design, causal direction cannot be established.

Routine self-reported PA assessment may provide a simple and practical component of lifestyle-based cardiometabolic risk stratification in primary care. Future longitudinal studies incorporating objective PA measures, dietary assessment, glycemic markers, medication data, and physiological biomarkers are warranted to clarify the pathways linking lifestyle behavior with diabetes risk.

## Figures and Tables

**Table 1:** Baseline characteristics of the study population according to self-reported PA levels (*n* = 863).

Variable	Low PA (*n* = 263)	Moderate PA (*n* = 341)	High PA (*n* = 259)	*p*-Value
Age (years)	59.45 ± 12.01	61.33 ± 11.85	59.09 ± 11.40	0.041
BMI (kg/m^2^)	29.15 ± 3.03	29.09 ± 2.91	29.20 ± 2.76	0.895
Waist circumference (cm)	99.63 ± 8.81	99.59 ± 8.70	100.86 ± 8.98	0.160
SBP (mmHg)	135.08 ± 8.52	135.03 ± 9.00	135.18 ± 8.58	0.977
DBP (mmHg)	82.51 ± 7.72	82.06 ± 7.75	82.07 ± 7.63	0.738
LDL-C (mmol/L)	3.31 ± 0.73	3.36 ± 0.73	3.16 ± 0.68	0.002
HDL-C (mmol/L)	1.30 ± 0.18	1.29 ± 0.18	1.31 ± 0.18	0.703
TG (mmol/L)	1.74 ± 0.44	1.75 ± 0.43	1.73 ± 0.44	0.939
Female, *n* (%)	118 (44.9%)	157 (46.0%)	121 (46.7%)	0.911
T2DM, *n* (%)	78 (29.7%)	57 (16.7%)	23 (8.9%)	<0.001
Smoking, *n* (%)	139 (52.9%)	201 (58.9%)	122 (47.1%)	0.015
Statin therapy, *n* (%)	136 (51.7%)	187 (54.8%)	115 (44.4%)	0.038

Values are presented as mean ± standard deviation or *n* (%). *p*-values were calculated using one-way ANOVA for continuous variables and *χ^2^* tests for categorical variables. PA, physical activity; BMI, body mass index; SBP, systolic blood pressure; DBP, diastolic blood pressure; LDL-C, low-density lipoprotein cholesterol; HDL-C, high-density lipoprotein cholesterol; TG, triglycerides; T2DM, type 2 diabetes mellitus.

**Table 2:** Prevalence of type 2 diabetes mellitus according to self-reported physical activity levels.

Self-Reported PA Level	Total, *n*	T2DM, *n* (%)	95% CI	Non-T2DM, *n* (%)
Low PA	263	78 (29.7%)	24.5–35.4	185 (70.3%)
Moderate PA	341	57 (16.7%)	13.1–21.0	284 (83.3%)
High PA	259	23 (8.9%)	6.0–13.0	236 (91.1%)
Overall	863	158 (18.3%)	15.9–21.0	705 (81.7%)

Values are presented as *n*, *n* (%), and 95% confidence interval (CI). The between-group difference in T2DM prevalence across PA categories was statistically significant (χ^2^ *p* < 0.001). PA, physical activity; T2DM, type 2 diabetes mellitus; CI, confidence interval.

**Table 3:** Association between self-reported PA levels and T2DM (logistic regression analysis).

Model	Comparison	OR	95% CI	*p*-Value
Model 1 (Crude)	Moderate PA vs. High PA	2.06	1.23–3.44	0.006
	Low PA vs. High PA	4.33	2.62–7.16	<0.001
Model 2 (Age- and sex-adjusted)	Moderate PA vs. High PA	2.06	1.23–3.45	0.006
	Low PA vs. High PA	4.31	2.61–7.13	<0.001
Model 3 (Fully adjusted)	Moderate PA vs. High PA	2.07	1.23–3.48	0.006
	Low PA vs. High PA	4.32	2.61–7.15	<0.001

Reference category: high PA. Model 1: crude model. Model 2: adjusted for age and sex. Model 3: adjusted for age, sex, BMI, smoking status, statin therapy, and systolic blood pressure. PA, physical activity; T2DM, type 2 diabetes mellitus; BMI, body mass index; OR, odds ratio; CI, confidence interval.

## Data Availability

The datasets used and/or analyzed during the current study are available from the corresponding author upon reasonable request.
